# Mutations in the *ND2* Subunit of Mitochondrial Complex I Are Sufficient to Confer Increased Tumorigenic and Metastatic Potential to Cancer Cells

**DOI:** 10.3390/cancers11071027

**Published:** 2019-07-21

**Authors:** Joaquín Marco-Brualla, Sameer Al-Wasaby, Ruth Soler, Eduardo Romanos, Blanca Conde, Raquel Justo-Méndez, José A. Enríquez, Patricio Fernández-Silva, Luis Martínez-Lostao, Martín Villalba, Raquel Moreno-Loshuertos, Alberto Anel

**Affiliations:** 1 Immunity, Cancer & Stem Cells Group, Department Biochemistry and Molecular and Cell Biology, Faculty of Sciences, Campus San Francisco Square, Aragón Health Research Institute (IIS Aragón), University of Zaragoza, E-50009 Zaragoza, Spain; 2 Aragón Health Research Institute (IIS Aragón), Center for Research in Biomedicine, E-50009 Zaragoza, Spain; 3 Department of Human Anatomy and Histology, Faculty of Medicine, Campus San Francisco Square, University of Zaragoza, E-50009 Zaragoza, Spain; 4 Carlos III National Center for Cardiovascular Research, 28029 Madrid, Spain; 5 GENOXPHOS Group, Department Biochemistry and Molecular and Cell Biology, Faculty of Sciences, Campus San Francisco Square, Biocomputation and Complex Systems Physics Institute (BIFI), University of Zaragoza, E-50009 Zaragoza, Spain; 6 Immunology Department, Lozano Blesa Clinical Hospital, E-50009 Zaragoza, Spain; 7 The National Institute of Biomedical Research (INSERM), Centre Hospitalier Universitaire de Montpellier, The University of Montpellier, The Institute for Regenerative Medicine and Biotherapy, 34090 Montpellier, France; 8 IRMB, CHU Montpellier, 34090 Montpellier, France

**Keywords:** mitochondria, dichloroacetate, complex I, *ND2*, metastasis, cybrids

## Abstract

Multiprotein complexes of the mitochondrial electron transport chain form associations to generate supercomplexes. The relationship between tumor cell ability to assemble mitochondrial supercomplexes, tumorigenesis and metastasis has not been studied thoroughly. The mitochondrial and metabolic differences between L929dt cells, which lost matrix attachment and MHC-I expression, and their parental cell line L929, were analyzed. L929dt cells have lower capacity to generate energy through OXPHOS and lower respiratory capacity than parental L929 cells. Most importantly, L929dt cells show defects in mitochondrial supercomplex assembly, especially in those that contain complex I. These defects correlate with mtDNA mutations in L929dt cells at the *ND2* subunit of complex I and are accompanied by a glycolytic shift. In addition, L929dt cells show higher in vivo tumorigenic and metastatic potential than the parental cell line. Cybrids with L929dt mitochondria in L929 nuclear background reproduce all L929dt properties, demonstrating that mitochondrial mutations are responsible for the aggressive tumor phenotype. In spite of their higher tumorigenic potential, L929dt or mitochondrial L929dt cybrid cells are sensitive both in vitro and in vivo to the PDK1 inhibitor dichloroacetate, which favors OXPHOS, suggesting benefits for the use of metabolic inhibitors in the treatment of especially aggressive tumors.

## 1. Introduction

During tumorigenesis, cells need to obtain nutrients for their rapid growth. Early studies by Otto Warburg [1] found that, even in the presence of oxygen, most cancer cells metabolize glucose by fermentation even though it generates ATP less efficiently than the aerobic processes of oxidative phosphorylation (OXPHOS), which takes place in mitochondria. The mitochondrial electron transport chain (mETC) is organized in the form of multiprotein respiratory complexes localized in the inner mitochondrial membrane, which accept electrons from NADH (complex I) or from FADH_2_ (complex II) for the creation of an electrochemical gradient which results in the generation of ATP in the ATP synthase complex or complex V. These complexes are not isolated in the inner mitochondrial membrane, but they rather associate in the form of supercomplexes in a dynamic way, depending on the energy state of the cell and on nutrient availability [2,3]. In fact, the capacity of mitochondria to organize the mETC in dynamic supercomplexes has been correlated with higher energetic efficiency [3,4]. 

Two hallmarks of cancer cells are escape from immune control and remodeling of energy metabolism [5]. Our group has described that metabolic changes are related with reduction of MHC-I expression, connecting both hallmarks [6,7]. Specifically, the generation of metastasis is associated with reductions in MHC-I expression and subsequent immune evasion [8]. Cancer cells suffer a stress-related metabolic remodeling when they lose the attachment to the culture plate, an event that could be related with metastasis initiation [9]. Interestingly, mutations in mtDNA that affect mitochondrial function have been linked to higher tumorigenic phenotypes [10,11,12,13,14], and in some cases to higher mitochondrial ROS generation [12]. 

In a previous study, we characterized a subline derived from murine L929 transformed fibroblasts that suffered matrix detachment, lost MHC-I expression and showed a glycolytic phenotype (L929dt cells, for “detached”; [15]). These phenotypes could be connected and the metabolic shift could be responsible for the increase in metastasis from solid tumors. For example, MHC-I loss in L929dt cells, correlates with lower sensitivity to cytotoxic T lymphocyte-mediated cytotoxicity and escape from immune surveillance [15]. In the present study, we have analyzed the mitochondrial and metabolic differences between L929dt and its parental cell line, observing defects in mitochondrial supercomplex formation and OXPHOS activities along with a shift to a more glycolytic metabolism. We demonstrate a higher in vivo tumorigenic and metastatic potential of L929dt cells compared with the parental cell line. This phenotype depends on mitochondrial DNA mutations in the *ND2* gene as shown by the generation of cybrid cell lines. In spite of their higher tumorigenic potential, cells harboring mitochondria with the *ND2* mutations (L929dt and the cybrid L929^dt^) are more sensitive both in vitro and in vivo to the PDK1 inhibitor dichloroacetate (DCA), which favors OXPHOS, than parental L929 cells. These data support the use of metabolic inhibitors to treat tumors with mitochondrial alterations. 

## 2. Results

### 2.1. Mitochondrial Supercomplex Assembly in L929 and L929dt Cells

Mitochondrial respiratory complexes associate in the inner mitochondrial membrane in the form of supercomplexes in a dynamic way [16], allowing cells to adapt better to their environment [3]. The impact of the cellular capacity to assemble mitochondrial supercomplexes in the context of tumor development or metastasis has not been studied in deep. We have compared supercomplex assembly in L929 cells and in its derived subline L929dt, which lost matrix attachment and showed signs of glycolytic metabolism in a previous study [15].

**Figure 1:**
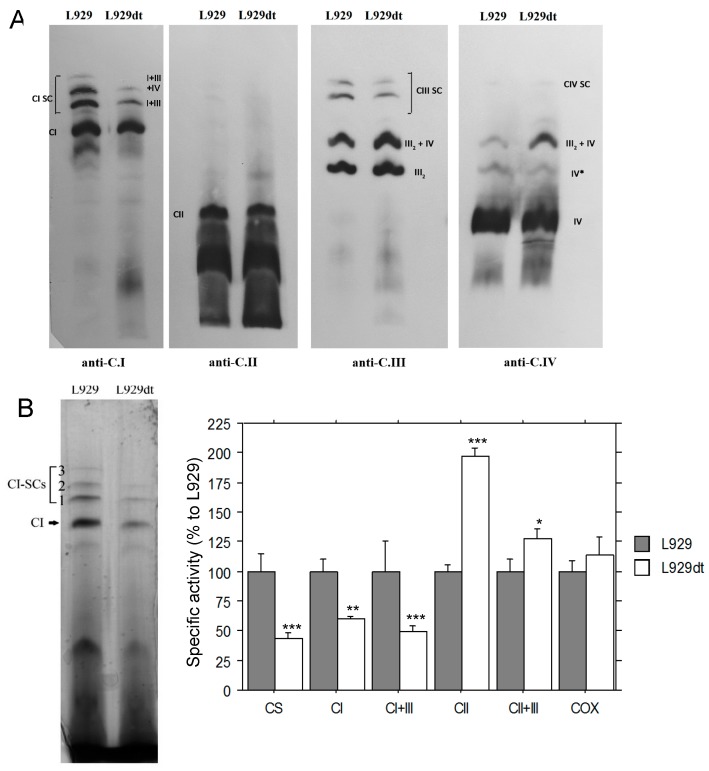
Mitochondrial supercomplex assembly and mitochondrial electron transport chain (mETC) complexes activity. (**A**) Mitochondria from L929 and L929dt cells were isolated, permeabilized using digitonin and mtETC complexes and supercomplexes were separated using blue native polyacrylamide gel electrophoresis (BNGE). Afterwards, proteins were transferred to a membrane and probed by immunoblot with monoclonal antibodies against complex I (anti-NDUFB6), II (anti-SDHA), III (anti-Core2) and IV (anti-Co1). The different supercomplexes (SC) and other associations are indicated on the blots: CI SC, supercomplexes that contain complex I: I + III or I + III + IV; CIII SC, supercomplexes that contain complex III; CIV SC, supercomplexes that contain complex IV. Data are representative of 6 different determinations. The amount of complex II in the same samples was used as loading control. (**B**) Left panel, BNGE followed by complex I in gel activity of the mitochondrial preparations solubilized with digitonin from L929 and L929dt cultured cells. Right panel, specific activities of mtETC complexes measured by spectrophotometry in mitochondria isolated from L929 and L929dt cells. All values are given as mean ± SD of the mean (*n* ≥ 3 in all cases). Asterisks indicate significant differences respect to L929 cells. *, *p* < 0.05; **, *p* < 0.02; ***, *p* < 0.01.

As shown in the immunoblot analysis of Figure 1A, the formation of supercomplexes containing complex I (I + III and I + III + IV) was greatly reduced in L929dt cells as compared with L929 cells. Levels of individual complex I were also partially reduced, although to a lesser extent than those of supercomplexes. This was also confirmed when analyzing supercomplex formation by immunoblotting against complex III. Complex II expression was similar in both types of cells, and free complex IV level was also similar. However, taking into account that more complex III is available, the formation of the supercomplex between complex III and IV was increased in L929dt cells. On the other hand, no difference was observed in complex V levels (Supplementary Figure S1).

### 2.2. Activity of Respiratory Complexes in L929 and L929dt Cells

The activity of the different complexes and supercomplexes was determined in biochemical assays as indicated in Materials and Methods. In the case of complex I, the activity was also determined in gel. As shown in Figure 1B, the activity of complex I, both free and associated with complex III, was substantially reduced in L929dt cells. It is interesting to point out that the reduction in activity was larger than the apparent observed reduction in its level by immunoblot analysis. On the contrary, the activity of complex II was clearly increased, and also partially that of complex II + III, indicating the existence of a compensatory response in the OXPHOS function of L929dt cells, that is defective, but not completely abrogated (Figure 1B). 

### 2.3. Metabolic Features of L929 and L929dt Cells

**Figure 2:**
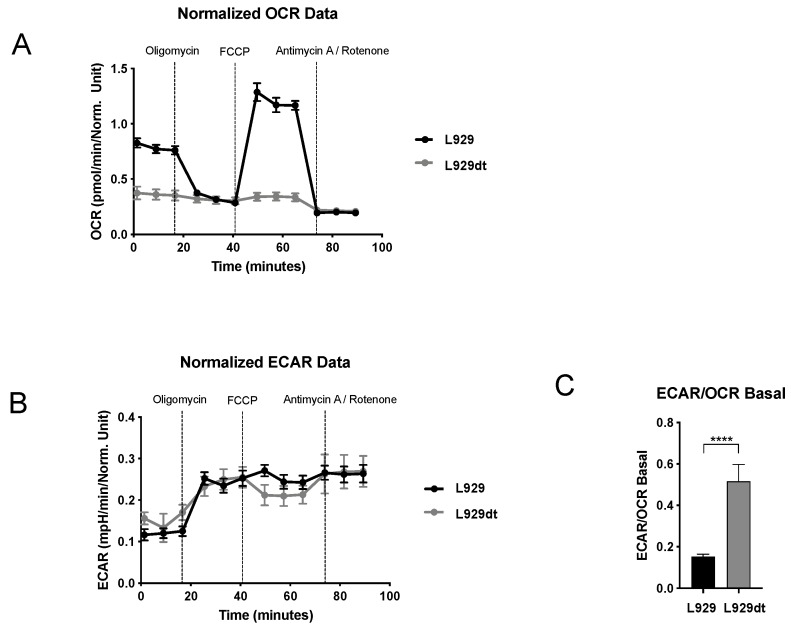
Analysis of mitochondrial respiration and glycolysis. (**A**) Oxygen consumption rate (OCR) and (**B**) extracellular acidification rate (ECAR) measurement upon sequential addition of oligomycin, FCCP, and rotenone+antimycin A in L929 (black dots) and L929dt (grey dots) cells using the Seahorse XF96 Extracellular Flux Analyzer. (**C**) ECAR to OCR ratio at basal respiration in both cell lines. Data are given as mean ± SEM with 8 independent assays per cell line. Asterisks indicate significant differences respect to L929 cells *p* < 0.0001 using non parametric Mann Whitney test.

To explore the impact on global cellular energy metabolism of the observed defects in supercomplex assembly and in respiratory complex activity, we tested oxygen consumption rate (OCR) and performed metabolic profiling measuring extracellular acidification rate (ECAR) comparatively in L929 and L929dt cells using the Seahorse technology. As shown in Figure 2, OCR was profoundly reduced in L929dt cells (Figure 2A). Regarding ECAR, although the normalized data did not show significant differences (Figure 2B), when data are shown as the ECAR/OCR basal ratio, L929dt cells showed an impairment in OXPHOS performance and a tendency towards a fermentative phenotype. This was further characterized by measuring endogenous and uncoupled respiration capacity of L929 and L929dt with an oxygen electrode (Supplementary Figure S2), and demonstrating that the duplication time in the presence of galactose was higher in L929dt cells compared with parental L929 cells (Supplementary Figure S3). 

### 2.4. Endogenous ROS Generation and Catalase Activity in L929 and L929dt Cells

**Figure 3:**
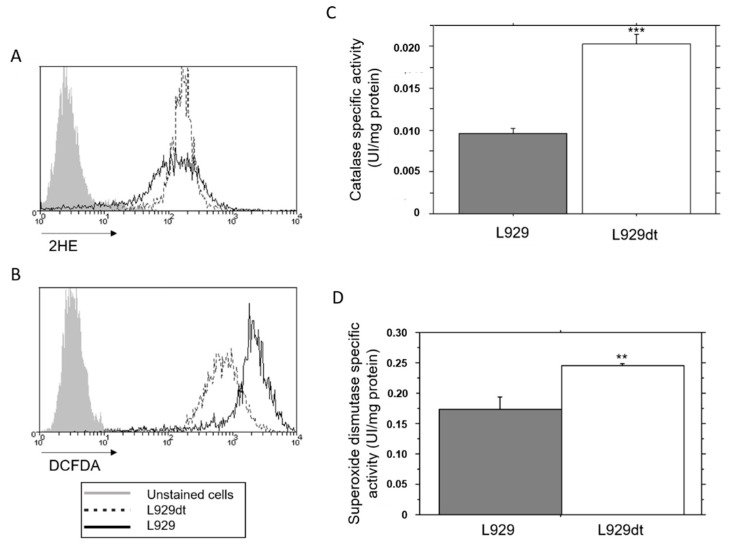
ROS formation, catalase and SOD activity in L929 and L929dt cells at the basal level. (**A**) Detection of superoxide anion by oxidation of dihydroethidium (2HE) and (**B**) oxidation of 2′,7′-dichlorodihydrofluorescein diacetate (DCF-DA) at the basal level in L929 (solid histogram) or L929dt cells (dashed histogram) were analyzed by flow cytometry. (**C**) Catalase specific activity was measured in cell homogenates as described in Materials and Methods. *n* = 3, ***, *p* < 0.001. (**D**) Mn-superoxide dismutase specific activity of L929 and L929dt cells in basal state was measured by spectroscopy, as indicated in Material and Methods. *n* = 3. ** *p* < 0.01.

The higher tumorigenic potential of cells with mitochondrial alterations has been associated in some studies with an increase in mitochondrial ROS generation [12]. We tested the levels of intracellular ROS in L929 and L929dt cells, using 2HE and DCF-DA. The fluorescence of 2HE is due to its specific oxidation by superoxide anion, while DCF-DA fluorescence reflects rather the levels of superoxide anion and also of H_2_O_2_. As shown in Figure 3A, superoxide anion levels were similar in both cell lines. However, DCF-DA fluorescence was higher in parental L929 than in L929dt cells (Figure 3B). Since superoxide anion levels were similar, this increased DCF-DA fluorescence should be due to a higher H_2_O_2_ content. H_2_O_2_ is generated in cells upon the activity of superoxide dismutase on superoxide anion, and the elimination of H_2_O_2_, still a powerful oxidant, is mediated by the enzyme catalase. To analyze this question, we performed enzymatic measurements of catalase activity and found that it was significantly higher in L929dt cells compared with L929 parental cells (Figure 3C), correlating with the reduction in H_2_O_2_ levels observed in these cells. In addition, total SOD activity was also substantially increased in L929dt cells (Figure 3D).

### 2.5. In Vivo Tumor Development of L929 and L929dt Cells

**Figure 4:**
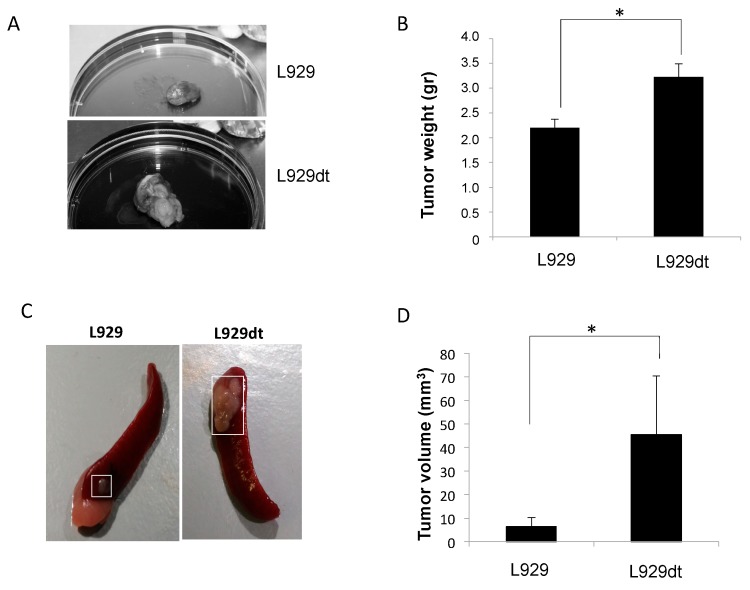
In vivo tumor development of L929 and L929dt cells in athymic mice. (**A**,**B**) 1 × 10^6^ L929 or L929dt living cells were injected s.c., respectively, in groups of 4 mice each. After three weeks, mice were sacrificed and tumors surgically excised. (**A**) Examples of the appearance and size of L929 or L929dt-derived tumors; (**B**) Mean ± SD of the tumor weights, expressed in grams. *, *p* < 0.05. (**C**,**D**) 125,000 L929 or L929dt living cells were injected intraesplenically, respectively, in groups of 5 mice each. After three weeks, mice were sacrificed and spleens surgically excised. (**C**) Examples of the appearance and size of L929 and L929dt-derived spleen tumors (squared areas); (**D**) Mean ± SD of tumor volumes, expressed in mm^3^. *, *p* < 0.05.

L929 cells do not induce tumors in syngeneic mice, probably due to an anti-tumor immune response, since they induce tumors in irradiated mice [17] and in athymic, immunodeficient mice [18]. We compared tumorigenicity of L929 and L929dt cells in the last model. We injected subcutaneously 1 × 10^6^ cells of each type in groups of mice and let them grow for 3 weeks. L929dt cells induced several tumor masses at the site of injection, while L929 cells induced smaller and individual tumors. Once resected, L929-derived tumors were compact and showed a homogeneous appearance. However, L929dt-derived tumors showed a more heterogeneous aspect, being clearly visible several tumor masses separated by connective tissue-type structures (Figure 4A). As shown in Figure 4B, the weight of L929dt-derived tumors was around 60% higher than the weight of L929-derived tumors, being this difference statistically significant. 

Trying to favor metastasis, we injected a smaller amount of tumor cells (125,000 viable cells per mice) in the spleen, a highly irrigated organ, of athymic mice. Although we did not detect metastasis in this experiment, we could observe again the higher tumorigenic potential of L929dt cells compared with L929 parental cells. As shown in the examples of Figure 4C, L929-derived tumors in spleens were small and round, while L929dt-derived tumors were bigger, and in several cases, multilobulated, similar to those observed after subcutaneous injection. As shown in Figure 4D, the mean volume of L929dt-derived tumors was significantly higher than that of L929-derived tumors (45.4 vs. 6.5 mm^3^). 

**Figure 5:**
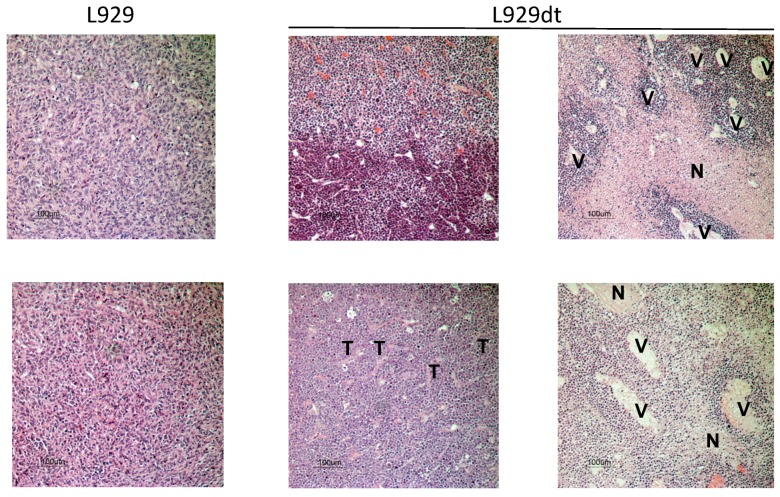
Hematoxilin/eosin staining in histological sections of L929 or L929dt-derived tumors in athymic mice. Paraffin tissue sections were stained with standard H&E coloration, as indicated in Material and Methods. Magnification was 100× in all images. N, necrotic areas; V, vessel-like structures; T, trabecular structures, all detected in L929dt-derived tumors, but not in L929-derived ones.

In the experiments shown in Figure 4A,B, hematoxylin/eosin (H/E) staining was also performed on resected tumors. Tumor tissue showed the typical fibroblastic appearance in the case of L929 cells, whereas L929dt cells showed a smaller and rounded morphology, in agreement with their tissue culture form (Figure 5).

In all L929-derived tumors, tissue organization was compact, with high cell density, indicating an elevated proliferation rate (see left panels). This feature was also seen in some L929dt-derived tumors (upper right panel). However, this high cell density was not homogeneous in the case of L929dt-derived tumors, showing different levels of compaction, with tumor zones less stained, probably corresponding to hypoxic and necrotic internal parts of the tumor (marked with an “N” in the rights panels). Moreover, structures similar to blood vessels surrounded in all cases by a very compact tumor mass (marked with “V” in the right panels), could be observed in L929dt-derived tumors, but not in any L929-derived tumor analyzed. This could indicate a higher capacity of L929dt-derived tumors to promote neovascularization, which could benefit tumor progression and metastasis. Finally, while in L929-derived tumors interstitial tissue was homogenously distributed in all tumor tissue, in those tumors derived from L929dt cells, interstitial tissue formed trabecular structures of fibrotic tissue (marked with “T” in the middle lower panel).

### 2.6. Determination of Mutations in Complex I Mitochondrial DNA in L929dt Cells Compared with Parental L929 Cells

**Figure 6:**
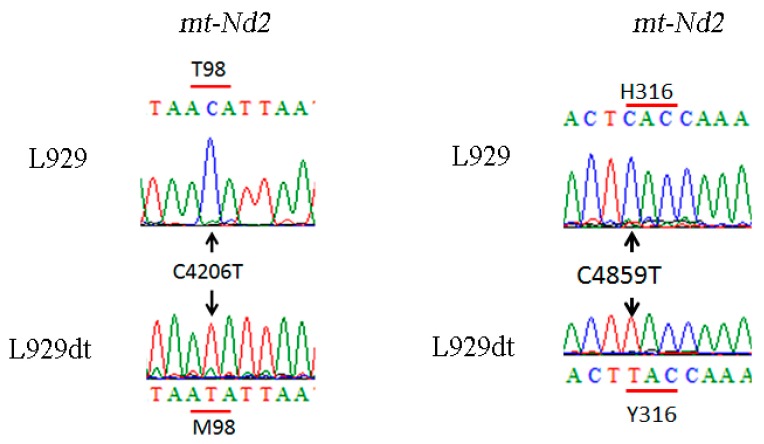
Characterization of complex I *mtDNA* mutations. Chromatograms showing the m.4206C > T and the m.4859C > T mutations within the *mt-Nd2* gene in L929dt cells.

Taking into account the defective activity and formation of supercomplexes containing complex I in L929dt cells (Figure 1), we decided to explore the appearance of specific mutations in the complex I components encoded in mtDNA by sequencing this genome. Two mutations were detected in the complex I subunit of mitochondrial origin *ND2* in L292dt cells compared with the parental L929 cell line (see Figure 6): (i) a C4859T change, that originates a change of a histidine for a tyrosine in the protein at position 316; and (ii) a C4206T change, that induces a methionine for a threonine change at position 98 of the protein. 

In addition, we found an increase in the number of adenine repetitions in a track located at position 9821 and corresponding to the mitochondrial Arg tRNA. While the number of repetitions is 10 in all copies of mtDNA in L929 cells, this number increases to 11 in part of mtDNA molecules (heteroplasmy) in L929dt cells (Supplementary Figure S4). 

### 2.7. Experiments with Cybrid Cells

To ascertain that the observed differences in the in vitro and in vivo phenotype of L929 and L929dt cells were due to the mutations in mtDNA described above, we generated cybrid cells, that is to say, L929 cells harboring mitochondria (and hence mtDNA) from L929dt cells (L929^dt^ cells), and conversely, L929dt cells expressing mitochondria of L929 cells (L929dt^L929^ cells), using previously optimized methods [16].

**Figure 7:**
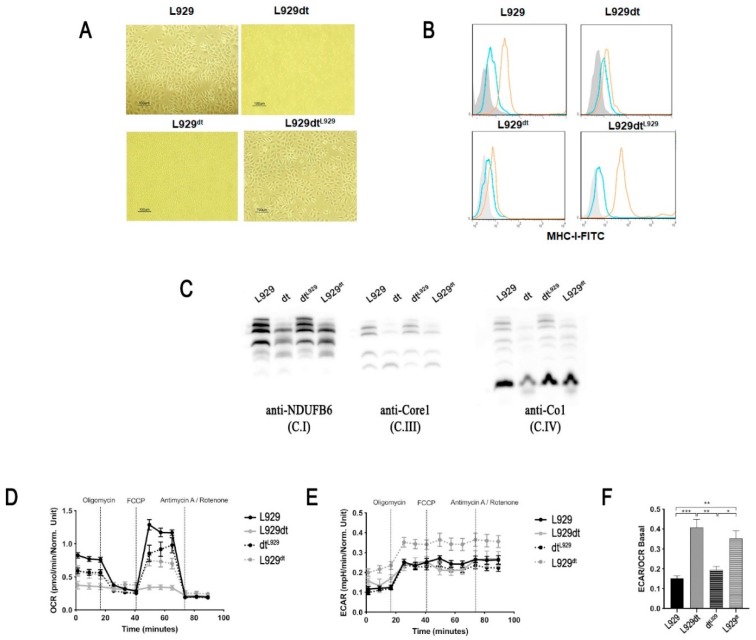
Properties of cybrid cells. L929^dt^ cybrid cells contain L929dt mitochondria on a L929 nuclear background and L929dt^L929^ cells contain L929 mitochondria on an L929dt nuclear background. (**A**) Phase contrast micrographs of cultures of L929, L929dt, or cybrid cells. (**B**) MHC-I expression on the surface of the indicated cells, determined by flow cytometry using a specific anti-H-2K^k^ antibody conjugated with FITC. Grey histograms, labeling of the cells alone; blue histograms, labeling with an irrelevant antibody of the same isotype and conjugated with FITC; orange histograms, labeling with the anti-H2-K^k^ mAb conjugated with FITC. (**C**) Supercomplex formation involving complex I, complex III or complex IV in the different cell types, determined by immunoblot in the same way as indicated in the legend of Figure 3. The amount of complex II in the same samples was used as loading control. (**D**) Oxygen consumption rate (OCR) and (**E**) extracellular acidification rate (ECAR) measurement upon sequential addition of oligomycin, FCCP, and rotenone + antimycin A in L929 (black dots and solid black lines), L929dt (grey dots and solid grey lines), L929^dt^ (grey dots and dashed lines) and dt^L929^ (black dots and dashed lines) cells using the Seahorse XF96 Extracellular Flux Analyzer. (**F**) ECAR to OCR ratio at basal respiration in the four cell lines, as indicated. Data are expressed as in Figure 2. *, *p* < 0.05; **, *p* < 0.01; ***, *p* < 0.001.

Regarding cellular morphological features, we observed that L929^dt^ cells detached from the culture plate, adopting a rounded shape and a smaller size, similar to that of L929dt cells. Conversely, L929dt^L929^ cells attached to the culture plate and adopted the typical fibroblastic shape of parental L929 cells (Figure 7A). In addition, L929^dt^ cells showed a dramatic reduction in surface MHC-I expression, arriving to the low expression level of L929dt cells. Conversely, L929dt^L929^ cells increased substantially MHC-I expression, reaching expression levels similar to those of parental L929 cells (Figure 7B). We also tested the pattern of mitochondrial supercomplex assembly in cybrid cells and compared it to that of L929 and L929dt cells. It was observed that the defective supercomplex assembly detected in L929dt cells was reproduced in L929^dt^ cells, with a drastic reduction in the amount of complex I-containing supercomplexes (SC I + III + IV and SC I + III). On the other hand, L929dt^L929^ cells showed a supercomplex assembly pattern that was very similar to that of parental L929 cells (Figure 7C). 

The metabolic comparison of the cybrid cell lines using the Seahorse technology showed clear differences in OCR measurements (Figure 7D), arriving to show a higher acidification capacity in the case of L929^dt^ cells in the ECAR measurements (Figure 7E). When showing the EACR/OCR data, the cybrid cell line L929^dt^ harboring mitochondria with the *ND2* mutations showed a clear tendency towards a fermentative phenotype compared with the line carrying normal mitochondria (Figure 7F). 

We also determined the mitochondrial superoxide anion basal level in these cells using the Mitosox probe, observing a certain increased level in cells containing mitochondria from L929dt cells with respect to cells with L929 mitochondria (Supplementary Figure S5). 

To explore the in vivo tumorigenic potential of cybrid cells, we designed an experiment to favor metastasis, injecting 250,000 viable cells in the spleens of athymic mice. After 3 weeks, we observed again that L929dt-derived tumors were bigger than those derived from L929 cells (mean of 645 vs. 198 mm^3^), and also that L929^dt^-derived tumors showed a substantially bigger size than tumors derived from L929dt^L929^ cells, that were in fact small (mean of 3105 vs. 8.55 mm^3^). If the size of tumors derived from cells expressing L929 mitochondria (L929 and L929dt^L929^ cells) is compared with the size of tumors derived from cells expressing L929dt mitochondria (L929dt and L929^dt^ cells), the difference is statistically significant (*p* = 0.05), with a mean of 1875 vs. 103 mm^3^, with data obtained from 6 mice in each group.

Regarding metastasis generation, we did not observe any metastasis in the six mice injected with cells expressing L929 mitochondria (L929 and L929dt^L929^ cells; see Table 1). However, in 1 out of 3 mice injected with L929dt cells, we observed one metastasis adjacent to the spleen, localized externally to the peritoneal membrane, with a much bigger size than the primary tumor in the spleen, and with a weight of 2 grs. In addition, in 2 out of 3 mice injected with L929^dt^ cells, we observed the generation of two big metastases in each of the mice. In both cases, one metastasis adjacent to the spleen was observed, similar to that found in mice 1 injected with L929dt cells, with a weight of 3 and 1.5 gr, respectively. Additionally, in one of these L929^dt^ injected mice, we observed another metastatic mass in the reproductive system, just between the two seminal channels, with a weight of 1.2 gr, while in the other mouse, we observed a metastasis in the stomach, that appeared grey and stiff, with a weight of 0.4 gr. These data clearly demonstrate the higher tumorigenic and metastatic potential of cells carrying mutated L929dt mitochondria. 

### 2.8. DCA Effect on L929 and L929dt and Cybrid Cells Proliferation, Cell Death and MHC-I Expression 

Dichloracetate is an inhibitor of PDK1 that forces cells to perform OXPHOS to produce ATP. In the case of tumor cells, with a tendency to use glycolysis for ATP production, DCA usually inhibits their growth, and can be considered as an adjuvant in tumor treatment [7]. The anti-tumor efficiency of DCA has been recently demonstrated in cells from leukemic patients. The mechanism of action of DCA in this context is partially dependent on the p53 status and implicates excessive ROS generation [19,20,21]. In our previous study, we demonstrated that DCA inhibited the growth of the leukemia EL4 and of L929 parental cells. It also induced cell death in their *ρ*^0^ sublines, devoid of mitochondrial DNA and, therefore, unable to generate ATP from the OXPHOS pathway [15].

**Figure 8:**
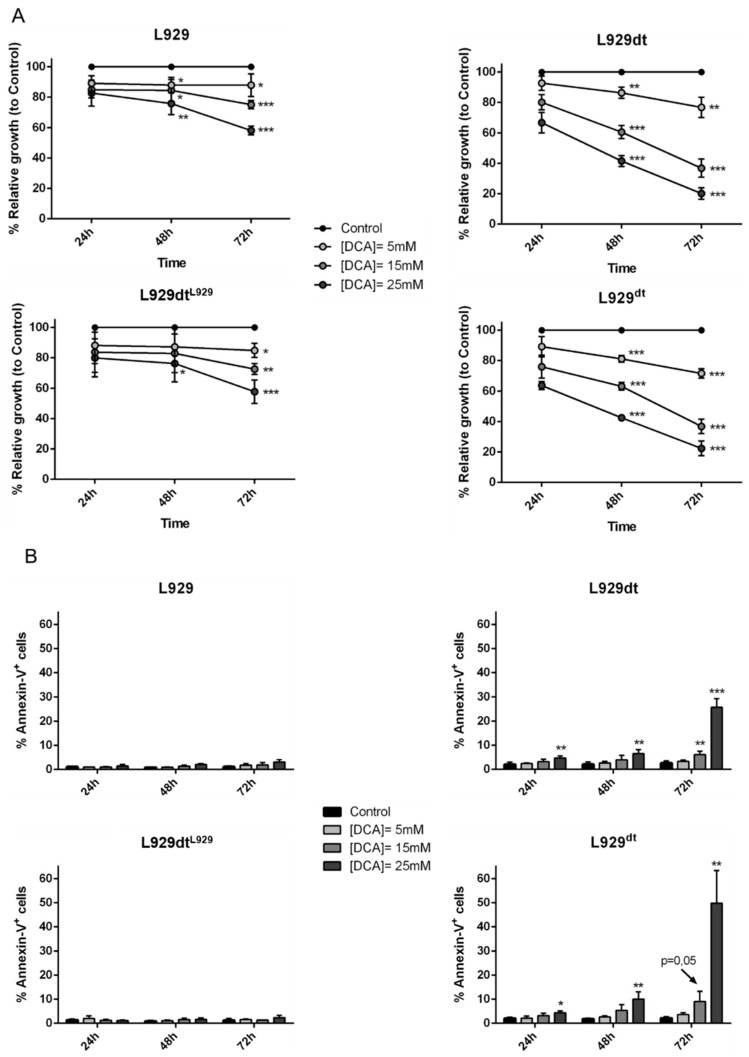
Effect of DCA on cell growth and cell death of the different cell lines studied. (**A**) Cells were supplemented or not (Control) during 72 h with the indicated concentrations of DCA, and every 24 h, cell growth was determined by the MTT reduction method and results expressed as percentage of cell growth relative to control cells. Results are the mean ± SD of three different experiments. *, *p* < 0.05; **, *p* < 0.02; ***, *p* < 0.01. (**B**) Cells were supplemented or not (Control) during 72 h with the indicated concentrations of DCA. Then, cells were stained with annexin-V-FITC and PS exposure, as a marker of apoptosis induction, was analyzed by flow cytometry. Results are the mean ± SD of three different experiments. *, *p* < 0.05; **, *p* < 0.02; ***, *p* < 0.01.

Here, we show that DCA partially inhibits L929 growth to a maximum of 40% after 72 h at the higher concentration used, 25 mM, while substantially inhibiting growth in L929dt cells, until 80% at 25 mM, and 60% at 15 mM (Figure 8A). In addition, while DCA did not induce cell death in L929 cells at any time or concentration, it induced small but significant cell death at 24 or 48 h of exposure in L929dt cells, arriving to almost 40% cell death at 72 h (Figure 8B). Remarkably, the same growing curves or cell death induction levels were obtained in cybrid cells that expressed the corresponding mtDNA: partial cell growth inhibition in the absence of cell death in L929dt^L929^ cells and substantial growth inhibition and even higher levels of cell death in L929^dt^ cells (Figure 8A,B).

**Figure 9:**
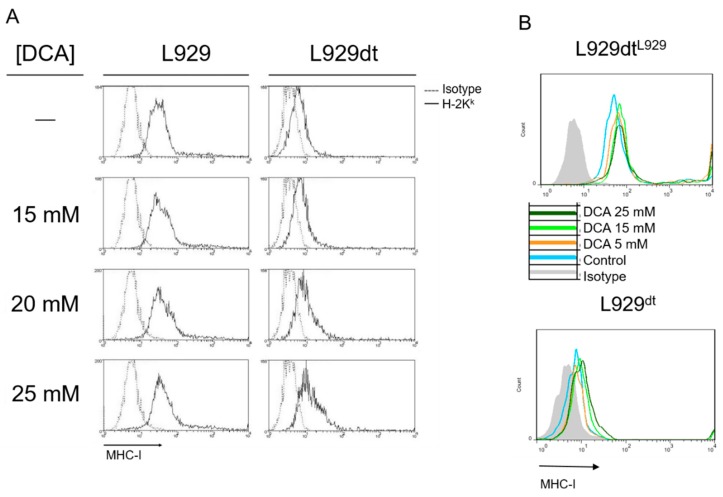
Effect of DCA on MHC-I expression. L929 or L929dt (**A**), and L929dt^L929^ or L929^dt^ cells (**B**) were supplemented or not (-) during 72 h with the indicated concentrations of DCA and MHC-I expression determined by flow cytometry using a specific anti-H-2K^k^ antibody conjugated with FITC. Dashed or grey histograms, labeling with an irrelevant antibody of the same isotype and conjugated with FITC; solid histograms, labeling with the anti-H2-K^k^ mAb conjugated with FITC. Data are representative of at least three different experiments.

In addition, surviving cells increased MHC-I expression in L929 and especially in L929dt cells, which had almost completely lost its expression (Figure 9A). This was again confirmed using cybrid cells: the high level of MHC-I expression observed in L929dt^L929^ cells was further increased after supplementation with DCA, while it notably increased the very low MHC-I expression exhibited by L929^dt^ cells (Figure 9B). Cells with low MHC-I expression are resistant to CTL-induced cytotoxicity, but DCA-induced increase in MHC-I expression would sensitize them to CTL, as demonstrated previously with L929dt cells [15]. This effect of DCA can have therapeutic implications, especially if this treatment is efficient on cells with high tumorigenic and metastatic potential.

**Figure 10:**
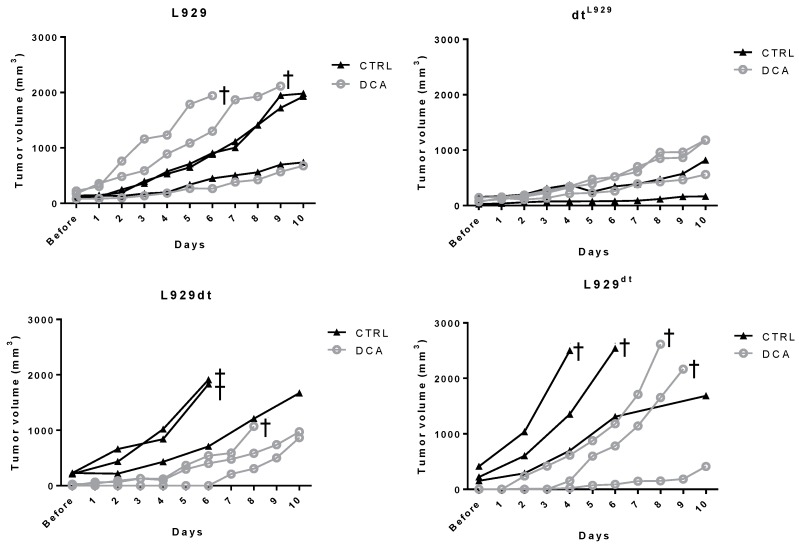
In vivo effect of DCA. 1 × 10^6^ L929, L929dt, L929dt^L929^ or L929^dt^ living cells were injected s.c., respectively, in groups of 6 mice each. When the tumor began to be detectable (20 mm^3^), each experimental group was divided in two, and three mice received intratumoral injections of 50 µL PBS every day during 10 days (Control, CTRL; black triangles) while the other three mice received injections of 25 mM DCA in 50 µL PBS with the same time schedule (DCA; grey circles). Data show tumor size during the 10 days of the treatments for each individual mouse. † indicates the sacrifice of mice due to ethical reasons before the end of the 10-day period.

Next, we performed an in vivo experiment using DCA treatment. We injected subcutaneously 1 × 10^6^ of each cell type in groups of athymic mice and when the tumor began to be detectable (20 mm^3^), we initiated the treatments. The time at which tumors began to be detected varied between the different tumor cell types: between 16 and 18 days after tumor injection for L929dt^L929^ cells; between 9 and 15 days for L929 cells; and 3 days in all mice injected with L929dt or L929^dt^ cells, demonstrating again the higher tumorigenicity of tumors with mitochondria from L929dt cells. As shown in Figure 10, while DCA treatment did not reduce the in vivo growth of tumors derived from L929 or L929dt^L929^ cells (upper panels), it was able to retard the rapid growth of tumors derived from L929dt or L929^dt^ cells (lower panels). The aggressive growth of these tumors obliged to sacrifice 2 of the mice in the control groups for ethical reasons 6 days after the initiation of the treatments, avoiding the calculation of statistical significances after the 10 day treatment period. However, at day 6, the retardation of growth induced by DCA in L929dt or L929^dt^-derived tumors was statistically significant (*p* < 0.05 in both cases). 

## 3. Discussion

In the last years, it has become clear that mitochondrial complexes are not isolated in the inner mitochondrial membrane, but that they rather associate forming supercomplexes in a dynamic way, depending on the cell type, the energy state of the cell and the availability of nutrients [2,3,4]. In fact, the capacity of mitochondria to organize the mETC in dynamic supercomplexes has been correlated with their higher energetic efficiency [3]. In the present study, we have analyzed the mitochondrial and metabolic differences between a cell line that spontaneously lost matrix attachment requirement to grow, i.e., L929dt cells, and their parental cell line and have correlated these changes with their tumorigenic potential. We found defects in the formation of supercomplexes depending on complex I, and a shift towards a higher activation of complex II. Strikingly, a similar shift in supercomplex dynamics has been reported recently during macrophage activation [22], showing that an apparent defect in mitochondrial respiration could be beneficial for certain cells in specific conditions. 

The analyses of the mutations present in L929dt mtDNA, compared with mtDNA of the parental cell line, pointed to a clustering of mutations in the *ND2* subunit of complex I, in agreement with the observed defects in supercomplex assembly. Ishikawa et al. [12] demonstrated a higher tumorigenic and metastatic potential in a murine lung tumor model associated with mutations in the complex I component *ND6*, but we did not find any difference in this gene between L929 and L929dt cells. Most previous studies on mtDNA mutations have been done in human tumors, and hence, the mutations detected in our murine tumors cannot be exactly the same mutations. Several mutations have been reported in the complex I component *ND2* in human mammary tumors [23,24], pancreatic cancer [25], oral cancer [26] and head and neck carcinomas [27,28]. Especially interesting is the report by Sun et al. [27], which describes that expression of the G4776A *ND2* mutation in cell lines induces loss of attachment to the culture plate, increase in ROS production, augmentation of HIF-1 expression and increase in PDK2 activity, augmenting also cell pyruvate levels. On the other hand, *ND4* mutations have been reported in acute myeloid leukemia [29] and in glioblastoma [30]. In addition, it has been demonstrated that a G11778A mutation in *ND4* was associated with a high tumorigenic potential [10]. The increase in the number of adenine repetitions in the track located at position 9821 and corresponding to the mitochondrial tRNA^Arg^ observed in L929dt cells could also result in an increase in mitochondrial ROS generation, as reported previously [31], although in this case it is apparently compensated by an increase in ROS-detoxifying activities. In addition, this change in tRNA^Arg^, in case of being detrimental, would most probably generate defects in all mitochondrial encoded complexes, and not only in complex I. A recent report has identified single nucleotide variants (SNVs) in mtDNA that correlated with metastasis generation in non-small cell lung cancer and colon cancer patients, although in this case the SNVs corresponded to the *ND1* subunit of complex I [32]. 

The fact that cybrids with L929dt mitochondria in the L929 nuclear background reconstituted all L929dt properties, clearly demonstrates that the mitochondrial mutations described are mostly responsible for the aggressive tumor phenotype. Conversely, cybrids with L929 mitochondria in the L929dt nuclear background retained L929 features, reinforcing the important role of mitochondria in final tumor phenotypes. Indeed, mitochondria regulate cell death via mitochondrial outer membrane permeabilization [33], and mitochondria from cancer cells frequently accumulate alterations and become dysfunctional for this mechanism, thus promoting cell survival. In addition, mitochondria are able to adapt their energetic metabolism depending on nutrient availability in the microenvironment, displaying anaplerotic pathways in order to optimize their surrounding resources during all stages of tumor progression [34]. Particularly, an enhanced Warburg phenotype, that is, a decreased OXPHOS function to obtain ATP, implies an increase in lactate production to regenerate intracellular NAD^+^. Lactate secretion to the tumor microenvironment also confers advantages to tumor cells by dampening the cytotoxic action of immune cells [35] and is related with worse prognoses in patients [36]. 

MHC-I loss could be related with the decrease in ERK5 expression observed in L929dt cells [15], which we had previously demonstrated to regulate MHC-I expression [37]. In fact, both expression and activity of ERK5 is increased by OXPHOS activity, which is defective in these cells [38]. A similar mechanism could be operative in the case of integrin expression, accounting for the attachment loss, although this has not been experimentally demonstrated. However, some data point to a role of ERK5 in integrin signaling, mediated by FAK activation [39]. 

The higher tumorigenic potential of cells with certain mitochondrial alterations has also been associated in some studies with an increase in mitochondrial ROS generation [12]. We have observed a somewhat increased level of mitochondrial superoxide anion in cells with mitochondria from L929dt cells, although the total cellular levels of superoxide anion were similar in L929 and L929dt cells, and the levels of H_2_O_2_ were reduced in L929dt cells compared with parental L929 cells. These reduced H_2_O_2_ levels correlated with a significant increase in catalase and MnSOD activity in L929dt cells. In this connection, it has been described that cellular stress caused by cell detachment from the extracellular matrix generates a deleterious increase in ROS generation in tumor cells, preventing metastasis generation [40]. However, successful metastatic cells present antioxidant adaptations that protect them from ROS generation, that were characterized in that study as increased levels of NADPH-generating enzymes in the folate pathway [40]. The increase in catalase and superoxide dismutase activity observed in L929dt cells could be an adaptation to oxidative stress by tumor cells that have developed high metastatic potential.

In this sense, we have found a higher in vivo tumorigenic and metastatic potential of L929dt or L929^dt^ cybrid cells compared with parental L929 cells or with the L929dt^L929^ cybrid. Cells with L929dt mutant mitochondria developed bigger tumor masses than cells expressing L929 mitochondria, associated with macroscopic findings such as the capacity for generating multilobulated primary tumors and tumor metastasis, adjacent and distant to the primary tumor. In addition, the observation of histological features typical of cells with a high growth rate, such as the presence of necrotic areas, mainly in the internal zone of the tumor, and high neovascularization, confirmed the higher tumorigenic potential of L929dt cells. 

Interestingly, in spite of their higher tumorigenic potential, cells harboring mutant L929dt mitochondria are more sensitive than cells with parental L929 mitochondria to the PDK1 inhibitor DCA, both in vitro and in vivo. Since DCA favors OXPHOS usage over glycolysis, it could have a specific effect in cancer cells that have accumulated mitochondrial defects and developed a more glucose-dependent metabolism as in the case here described. In addition, DCA-induced increase in MHC-I expression would sensitize tumors to the action of CTL. Thus, this and other metabolic inhibitors could be potential tools in the treatment of especially aggressive tumors, alone or in combination with chemotherapy and/or immunotherapy. 

## 4. Materials and Methods

### 4.1. Cells and Mice

Immune-deficient athymic mice, Swiss nu/nu strain, six-week old males, were used in the present study. Mice were purchased from Charles River (Wilmington, MA, USA) or from Janvier Labs (Le Genest-Saint-Isle, France). All mice were maintained in a specific pathogen-free facility and were group-housed with controlled lighting and temperature. Animals were on an ad libitum irradiated diet and autoclaved water. All procedures were carried out under Project License P17/16 approved by the Ethic Committee for Animal Experiments from the University of Zaragoza. The care and use of animals were performed accordingly with the Spanish Policy for Animal Protection RD53/2013, which meets the European Union Directive 2010/63 on the protection of animals used for experimental and other scientific purposes.

The mouse fibroblast cell lines L929 and L929-derived L929dt were cultured in high glucose DMEM (GIBCO^TM^) medium supplemented with 10% heat-inactivated FBS (Sigma, Saint Louis, MO, USA), penicillin/streptomycin (Pan Biotech, Aidenbach, Germany) and GlutaMAX (Gibco, Waltham, MA, USA) (complete DMEM medium).

### 4.2. Flow Cytometry Determination of MHC-I Surface Expression, Apoptosis Induction and ROS Production

For the determination of MHC-I surface expression, 1 × 10^5^ cells were stained for 30 min at 4 °C with anti H-2K^k^-FITC or isotype control in 100 μL PBS 5% FBS. Both antibodies were obtained from BD Pharmingen. Cells were washed and analyzed in the flow cytometer, gating previously the viable population using light scattering data. 

For the measurement of apoptosis induction, 1 × 10^5^ cells were stained for 15 min at room temperature with Annexin-V-FITC (Immunostep), which binds to the phosphatidylserine exposed in the cell surface, in annexin-binding buffer (140 mM NaCl, 2,5 mM CaCl_2_, 10 mM HEPES/NaOH, pH 7.4). Cell suspension was diluted to 200 μL with the corresponding buffer and analyzed by flow cytometry.

For the determination of ROS production, 1 × 10^5^ cells were stained for 30 min at 37 °C with either 2 μM DHE (dihydroethidium, Life Technologies, Carlsbad, CA, USA) or 20 μM H_2_DCF-DA (2′,7′-dichlorodihydro-fluorescein diacetate, Life Technologies). While DHE staining only allows superoxide anion detection, H_2_DCF-DA staining also measures H_2_O_2_ generation and other ROS species [41,42]. After incubation, cells were washed with PBS and analyzed in the flow cytometer.

Samples were analyzed using a FACSCalibur flow cytometer and CellQuest software (both of them from BD Biosciences, Franklin Lakes, NJ, USA).

### 4.3. Mitochondrial Superoxide Production Analysis

For mitochondrial ROS production, cells were incubated with MitoSOX (5 µM) for 30 min at 37 °C, before assessment in a FACSCalibur (BD Biosciences) cytometer. Data were analyzed using FLowJo software.

### 4.4. Cell Viability Assays

Relative cell growth was measured by the method of Mosmann modified for microplates [43]. Briefly, 3 × 10^5^ cells/mL were cultured in 96-well flat-bottomed plates for 24–72 h. After the indicated time, each well was mixed with 10 μL of a MTT dye solution (3-(4,5-dimethylthiazol-2-yl)-2,5 diphenyltetrazolium bromide, 5 mg/mL in PBS). Alive cells contain enzymes that can reduce this reactive to an insoluble purple formazan crystal. After 2–3 h incubation, all formed crystals were centrifuged and solubilized in isopropanol. Finally, the absorbance of each well was measured in a microplate reader (Dynatech, Pina de Ebro, Spain). Results are expressed as the percentage of relative growth of cells exposed to DCA in comparison with non-treated cells.

### 4.5. Supplementation with DCA or Galactose

In certain experiments, cells were grown in complete DMEM medium in the presence or absence of DCA (Sigma) at different concentrations (5–25 mM) and at several times (24–72 h). Relative cell growth was measured by the modified Mosmann’s method and MHC-I surface expression by flow cytometry, as indicated above. 

To measure their growth capacity in galactose containing medium, cells were cultured in glucose-free DMEM medium supplemented with 10% heat-inactivated FBS (Sigma), 0.9 g/L galactose and 0.11 g/L sodium pyruvate. Cell growth was followed for 96 h, performing cell counts at daily intervals. Duplication time (D_T_) of cells in glucose or galactose was calculated by representing the number of cells vs time in a graph and adjusting the values to an exponential curve as: *N* = a × 10^bt^ so that D_T_ was obtained using the formula: D_T_ = Log 2/b.

### 4.6. Blue Native Polyacrylamide Electrophoresis

Mitochondria were isolated from cultured cell lines according to Schägger [44], with some modifications [2]. Digitonin-solubilized mitochondrial proteins (100 μg) were separated on blue native gradient gels (3–13% acrylamide). After electrophoresis, the gels were further processed for immunoblotting or in gel complex I activity.

### 4.7. Analysis of Mitochondrial Supercomplex Assembly by Immunoblot

After electrophoresis, the complexes were transferred onto PVDF filters, and then membranes were blocked with TBS-T buffer (10 mM Tris / HCl, pH 8.0, 0.12 M NaCl, 0.1% Tween-20, 0.05% thimerosal) containing 5% non-fat milk. Antibodies in TBS-T containing 2% non-fat milk against complex I, anti-NDUFB6 (Molecular Probes, Eugene, OR, USA); complex III, anti-core2 (MitoSciences, Eugene, OR, USA); complex IV, anti-COI (MitoSciences), and complex II, anti-SDHA (Molecular Probes) were used to incubate membranes. After that, membranes were washed again with TBS-T and incubated with 0.2 μg/mL of the secondary antibody labeled with corresponding peroxidase (Sigma). Finally, proteins were revealed with the Pierce ECL Western Blotting Substrate (Thermo Scientific, Waltham, MA, USA). The amount of complex II in the same samples was used as a validated loading control. 

### 4.8. In Gel Complex I Activity

For the determination of in gel complex I activity, a solution of Tris-HCl 0.1 M pH 7.4, NADH 10 mM and nitroblue tetrazolium (NBT) 1 mg/mL was added on the surface of the polyacrylamide gel. Complex I activity was revealed by a blue precipitation in the correspondent bands.

### 4.9. OXPHOS Performance

In order to assess cellular metabolic functions such as oxidative phosphorylation and glycolysis, oxygen consumption rate (OCR) and extracellular acidification rate (ECAR) measurements of cultured cells were performed using the XF96 Extracellular Flux Analyzer (Seahorse Biosciences, North Billerica, MA, USA). Both parameters were measured in basal conditions (Seahorse media with 1 mM pyruvate, 2 mM glutamine, 1 M glucose at pH 7.4 was added 30 min before the analysis and cells were incubated at 37 °C in the incubator without CO_2_) and after the sequential addition of oligomycin (to inhibit complex V), FCCP (to uncouple respiration and ATP synthesis), and rotenone/antimycin A (which inhibit complex I and complex III respectively). Final drug concentrations were 1 μM. For every replicate, 30,000 cells/well were plated when adherent cells were used, and 50,000 cells/well when L929dt or L929^dt^ cells immobilized on a poly-l-lysine coated surface were analyzed. After every assay the number of cells was determined and OCR and ECAR data were normalized by the number of viable cells using CyQuant (Thermo Fisher, Waltham, MA. USA). 

O_2_ consumption in intact or digitonin-permeabilized cells was determined with an oxytherm Clark-type electrode (Hansatech, Pentney, UK) as previously described [45] with small modifications [46].

Mitochondria from cell lines were isolated as described [47]. Rotenone sensitive NADH-dehydrogenase activity (CI activity), succinate dehydrogenase activity (CII activity), cytochrome c oxidase activity (COX, CIV activity), NADH cytochrome c oxido-reductase activity (CI + III activity), succinate (G3P) cytochrome c oxido-reductase activity (CII + III or G3PDH + III activities) and citrate synthase (CS activity) were measured as previously described [3]. 

### 4.10. Analysis of Mitochondrial DNA Mutations

Total DNA from cell lines was extracted using standard procedures. Overlapping segments of 800–1000 bp long PCR fragments covering the mtDNA-encoded complex I subunits and *mt-Tr* genes were amplified by PCR with the appropriate oligodeoxynucleotides (Supplementary Table S1). The purified double stranded fragments were sequenced using nested primers (Supplementary Table S2). All the primers were designed using the reference sequence (NC_005089) [47].

Confirmation of the C4206T and C4859T mutations within mt-Nd2 gene was achieved by RFLP analysis. First, two DNA fragments containing the mutations sites, were amplified by PCR using the primers indicated in Supplementary Table S3. The presence of the 4206T version generates a restriction site for SspI whereas the mutant 4589T version eliminates a recognition site for HphI.

### 4.11. Generation of Cybrid Cells

Transmitochondrial cell lines were obtained by two different methodologies. To generate L929dt^L929^ cybrid cells, L929dt cells (nucleus donors) were pretreated with a lethal dose of rhodamine 6 G and rescued by fusion with L929 cytoplasts as previously described [16]. Due to the non-adherent nature of L929dt cells, an alternative methodology was necessary for the generation of L929^dt^ cybrids. Briefly, L929dt isolated mitochondria were fused with *ρ*^0^ L929 cells in the presence of PEG as previously described for the use of human platelets as mitochondria donors [48].

### 4.12. Determination of Catalase and Superoxide Dismutase Specific Activity

Catalase activity was assayed in total cellular homogenate as described [31]. Briefly, the absorbance decrease at 240 nm was measured in a medium containing 20 mM H_2_O_2_ and 10 mM potassium phosphate buffer (pH 7). One unit of the enzyme was defined as 1 mmol of H_2_O_2_ consumed per minute. Superoxide dismutase (SOD) specific activity was assayed as well in total cellular homogenate. SOD ability to stop nitro-blue tetrazolium (NBT) reduction by O_2_^−^ was measured by absorbance decrease at 560 nm in a medium containing 2.3 µM riboflavin, 60 mM potassium phosphate buffer (pH 7), 6.4 mM EDTA and 23.5 µM tetramethylethylenediamine (TEMED). Both activities were measured with a UV 500 spectrophotometer (Unicam). We confirmed that the detected activity was mainly due to mitochondrial MnSOD by complete inhibition with sodium cyanide. Specific activity is reported as units per milligram of protein estimated by the Bradford method [49].

### 4.13. In Vivo Tumor Development Experiments

For in vivo tumor development experiments, 1 × 10^6^ cells were injected subcutaneously in 0.1 mL PBS. Tumor growth evolution and mice wellness were followed for 3 weeks, when mice were sacrificed. Tumor growth was analyzed by measuring the tumor daily with a caliper of precision. To calculate the tumor volume, the width (A) and length (L) of the tumor were measured, and the following formula was applied: V = L × A^2^/2. At the end of the experiment, solid tumors were surgically excised, their size measured, weighted, and then fixed in 4% formaldehyde and embedded in paraffin for histochemistry analysis. In the experiments using DCA, treatments were initiated when the tumor began to be detectable (20 mm^3^), with control mice receiving intratumoral injections of 50 µL PBS every day during 10 days while treated mice received injections of 25 mM DCA in 50 µL PBS with the same time schedule. 

To favor metastasis generation, we developed in vivo experiments in which intra-splenic injection were performed. Mice were anesthetized with isoflurane (4% induction, 2% maintenance). A 1.5 cm lateral incision was made in the left subcostal flank of each mouse and spleen externalized through the incision. With the aid of a 50 µL gastight syringe (1705 LT SYR, Hamilton, Reno, NV, USA) equipped with a 27-gauge needle, 125,000 or 250,000 viable cells in 25 µL PBS were injected into the spleen. The peritoneum and the margins of the incision were then sutured. After 3 weeks, mice were sacrificed and spleens surgically removed to measure the size of primary tumors, as indicated above. Proximal or distal metastasis were also localized in the animals, surgically removed, measured and weighted. Finally, they were fixed in 4% formaldehyde and embedded in paraffin as indicated above. 

### 4.14. Histological Studies

Tissue samples processed were performed at the Microscopy and Anatomic Pathology Core Unit of the Institute for Health Sciences of Aragon (Zaragoza, Spain). Tissue samples were collected, trimmed and fixed in 4% neutral buffered formaldehyde solution and then processed in a rapid tissue processor (X-PRESS ×50 processor, Sakura, Chiba, Japan) until paraffin embedded. Once paraffin blocks were made, 2.5 μm sections were cut with a rotation microtome (Leica RM2255) and paraffin sections were taken on glass slides. Slides were air dried at 37 °C, and for hematoxylin–eosin staining, they were deparaffinized in xylene for 10 min, rehydrated in a grades series of ethanol and distilled water for 5 min. The nuclei of the cells in tissue sections were stained by immersing in Carazzi’s Hematoxylin solution (Panreac) for 5 min and washed. Cell cytoplasm was stained by immersing in 1% of eosin yellowish hydroalcoholic solution (Panreac) for 15 min and dipped once quickly in 70% of ethanol for 30 s. After eosin staining, sections were dehydrated by immersion in ascending alcohol solutions for 15 s/each. Finally, all sections were cleaned by xylene for 15 s and mounted with a coverslipping reagent (Leica CV Mount) using glass coverslips. 

### 4.15. Statistical Analysis

Differences between tumor weights in L929/L929dt solid tumors in mice were assessed using the Student t test. For the rest of the experiments, comparisons were made by Fisher’s PLSD post hoc test. Data were analyzed with StatView (Adept Scientific, Luton, UK) or with the GraphPad Prism 8.1.2 software (California, CA, USA) for the Seahorse experiments. In all cases, differences were considered statistically significant at *p* < 0.05. 

## 5. Conclusions

In this study, we demonstrate that mutations in the *ND2* subunit of the mitochondrial complex I are sufficient to confer a higher tumorigenic and metastatic potential, causing also a glycolytic shift and defects in the assembly of supercomplexes containing complex I. In spite of this, cells harboring mutant mitochondria are more sensitive than cells with parental mitochondria to the PDK1 inhibitor DCA, both in vitro and in vivo. Thus, this and other metabolic inhibitors could be useful in the treatment of especially aggressive tumors, alone or in combination with chemotherapy and/or immunotherapy.

## Figures and Tables

**Table 1:** Metastasis detection in mice injected in the spleen with 250,000 L929 or L929dt viable cells or with the cybrids L929dt^L929^ or L929^dt^. Groups of 3 mice were injected intraesplenically with 250,000 living cells per mice of the 4 different cell lines indicated, respectively. The number, localization and weight of the metastasis detected in each mouse is indicated, “-“ means that no metastasis was detected.

	Metastasis Detection			
	L929	L929dt	dt^L929^	L929^dt^
**Mouse 1**	---	1 (adjacent, 2 gr)	---	2 (adjacent, 3 gr; Stomach, 0.4 gr)
**Mouse 2**	---	---	---	---
**Mouse 3**	---	---	---	2 (adjacent, 1.5 gr; Seminal, 1.2 gr)

## Supplementary Materials

The following are available online at https://www.mdpi.com/2072-6694/11/7/1027/s1, Figure S1: Expression of complex V (α-βF_1_ATPase) in L929 and L929dt cells, Figure S2: Oxygen consumption of L929 and L929dt cells, Figure S3: Duplication time of L929 and L929dt cells, Figure S4: Alanine repetitions in L929 and L929dt cells, Figure S5: Determination of the amount of mitochondrial superoxide anion in basal conditions in L929, L929dt, L929dt^L929^ and L929^dt^ cells, Table S1: PCR primers, Table S2: Sequencing primers, Table S3: RFLP analysis.
