# Supplementary material for: Mutations in the ND2 Subunit of Mitochondrial Complex I Are Sufficient to Confer Increased Tumorigenic and Metastatic Potential to Cancer Cells
Source: Cancers (Basel). 2019 Jul 21;11(7):1027. doi: 10.3390/cancers11071027 (PMC6678765; doi:10.3390/cancers11071027)
Supplement: Supplementary file 1 [file cancers-11-01027-s001.pdf]

cancers

Supplemental Materials

Mutations in the *ND2* Subunit of Mitochondrial Complex I Are Sufficient to Confer Increased Tumorigenic and Metastatic Potential to Cancer Cells

Joaquín Marco-Brualla, Sameer Al-Wasaby, Ruth Soler, Eduardo Romanos, Blanca Conde, Raquel Justo-Méndez, José A. Enríquez, Patricio Fernández-Silva, Luis Martínez-Lostao, Martín Villalba, Raquel Moreno-Loshuertos and Alberto Anel

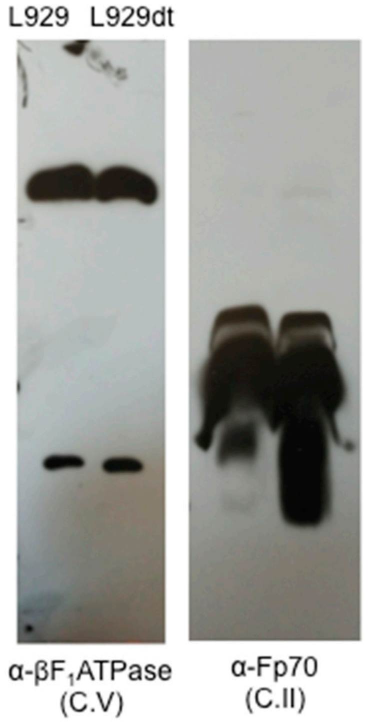

Figure S1. Expression of complex V (α-βF_1ATPase) in L929 and L929dt cells. Mitochondria from both cell lines were isolated and permeabilized using digitonin and mtETC complexes and supercomplexes were separated using BNGE. Afterwards, proteins were transferred to a membrane and probed with monoclonal antibodies for (anti-β-F1ATPase) and CII (anti-Fp70). Western-Blotting of complex II (α-Fp70) was used as loading control.

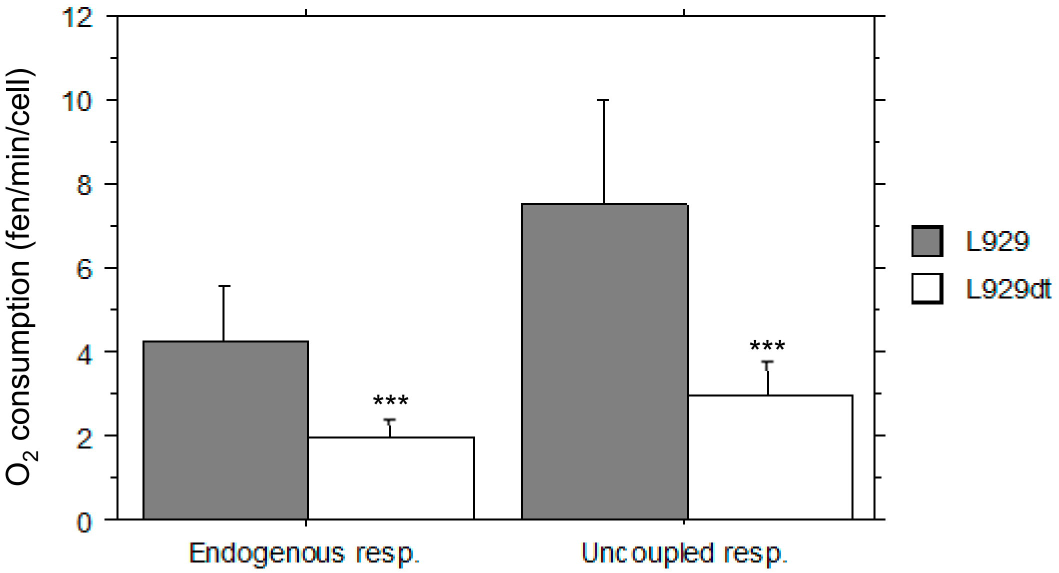

Figure S2. Oxygen consumption of L929 and L929dt cells, expressed as endogenous and uncoupled respiration. Data shown correspond to mean +/− SD of n ≥ 6. *** $p < 0.001$.

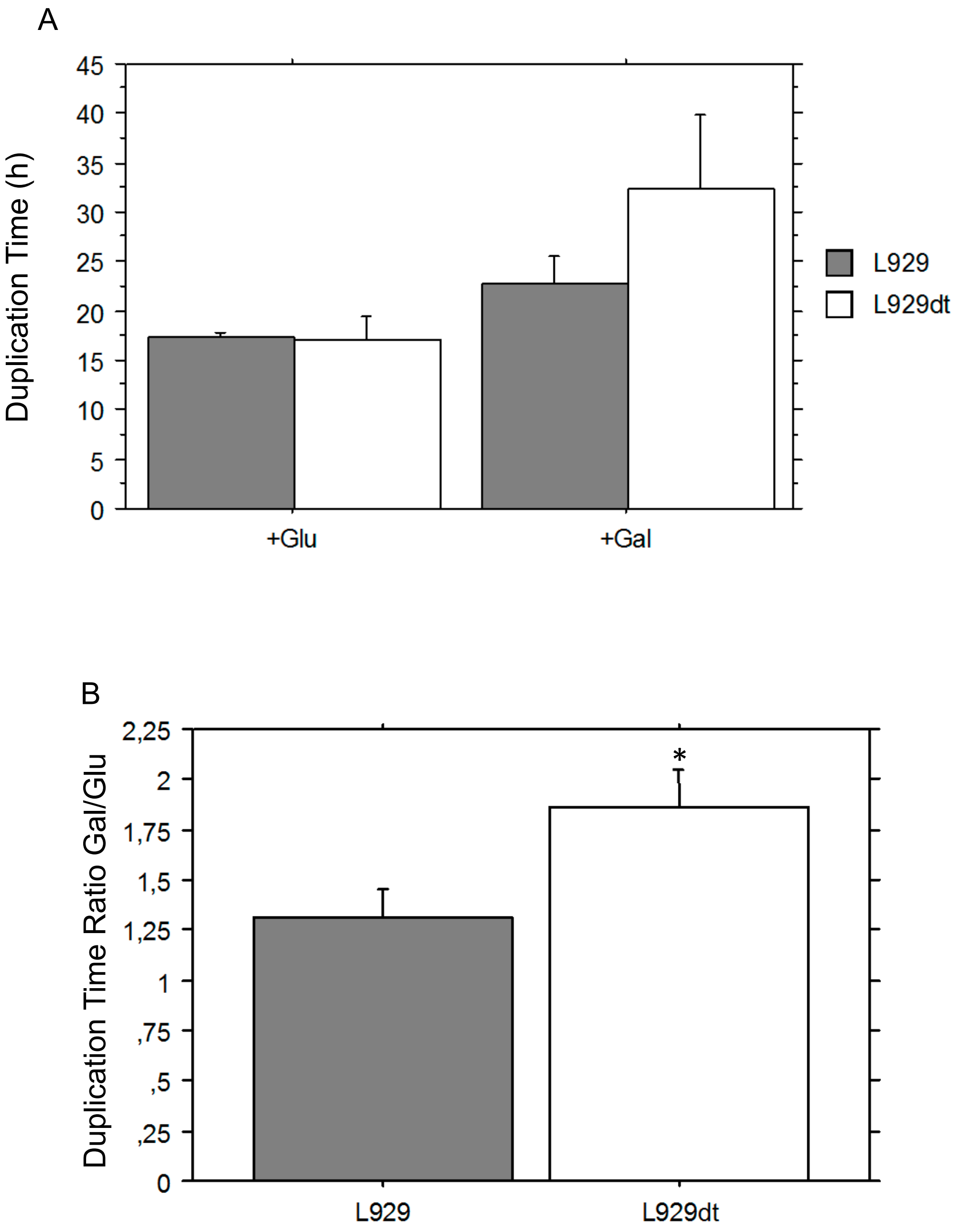

Figure S3. Duplication time of L929 and L929dt cells, in the presence of (A) glucose (Glu) or galactose (Gal); and (B) calculated as Duplication Time ratio of Glu/Gal, showed as the mean +/− SD of n = 3 (L929) and n = 2 (L929dt). * $p < 0.05$.

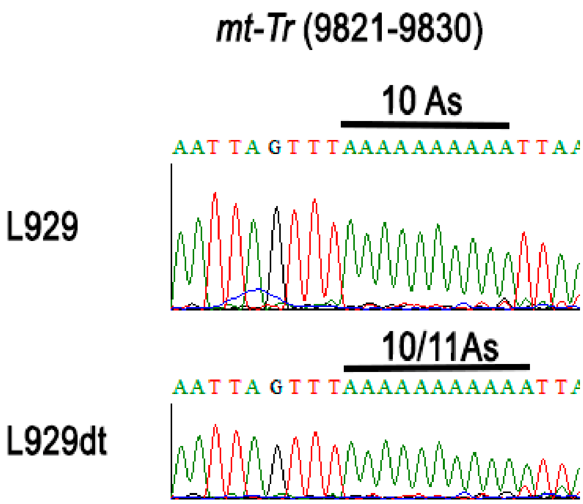

Figure S4. Alanine repetitions in L929 and L929dt cells. Chromatograms showing the heteroplasmic insertion of an adenine in the polymorphic adenine track of the *mt-Tr* gene in L929dt cells.

Table S1. PCR primers.

Fragment	Oligo name	Position (NC_005089)	Sequence	Fragment size
1	1F	1918–1939	ACAAGAACCCCGCCTGTTTACC	1607
	1R	3504–3525	AGTTAGTTGAGTAGAGTTCTGG	
2	2F	3269–3291	ATTACTTCTGCCAGCCTGACCCA	1522
	2R	4802–4821	GTTAGTGAAGTGGAATAAAT	
3	3F	3862–3883	AAGCTATCGGGCCCATACCCCG	1343
	3R	5184–5205	CTCTACTAAGACTTCTACCGCC	
4	4F	9072–9093	CGAAACCACATAAATCAAGCCC	1973
	4R	11024–11045	GTGGCTAACTGAGGAGTAGGCG	
5	5F	10803–10824	CACCTATGACTACCAAAAGCCC	1361
	5R	12186–12164	TTCCCACCCCTTCTCAGCCAATG	
6	6F	11703–11724	TAGGAACCAAAAACCTTGGTGC	1221
	6R	12903–12924	TTAGGTCTTTTGAGTAGAACCC	
7	7F	12385–12406	GCCTATTAATCGCAGCTACAGG	1844
	7R	14208–14229	ATGAAATGTTGGATGGGGCAGG	

Table S2. Sequencing primers.

Fragment	Primer name	Position	Sequence
1	1a	2572–2593	GGACAAGAGAAATAGAGCCACC
	1b	3010–3031	TTCCCCTACCAATACCACACCC
2	2a	3269–3290	ATTACTTCTGCCAGCCTGACCC
3	3a	3862–3883	AAGCTATCGGGCCCATACCCCG
	3b	4448–4469	ATAGCCTATTCATCAATTGCCC
4	4a	9301–9322	ACTTCGGATTTGAAGCCGCAGC
	4b	9745–9766	CAATTCTATCTCTAGGCCTAGC
	4c	10326–10347	AATATATTCTCCTCAGACCCCC
5	5a	10803–10824	CACCTATGACTACCAAAAGCCC
	5b	11460–11481	CGAGAACTAACACTAATAGCCC
6	6a	12000–12021	ATCCTGTTTACATCTGTAGCCC
	6b	12255–12276	ATCCTCTATAACCGCATCGGAG
7	7a	12852–12873	TCATGCCTAGTAATCGGAAGCC
	7b	13423–13444	CCATCCCAAAATCCACCTCAAC

Table S3. RFLP analysis.

Mutation	Forward primer position	Reverse primer position	Restriction enzyme name	L929 fragments (4206/4859C)	L929dt fragments (4206/4859T)
C4206T	3862–3884	4236–4258	SspI	306+118 bp	306+79+39 bp
C4859T	4448–4470	5184–5205	HphI	433+304 bp	737 bp

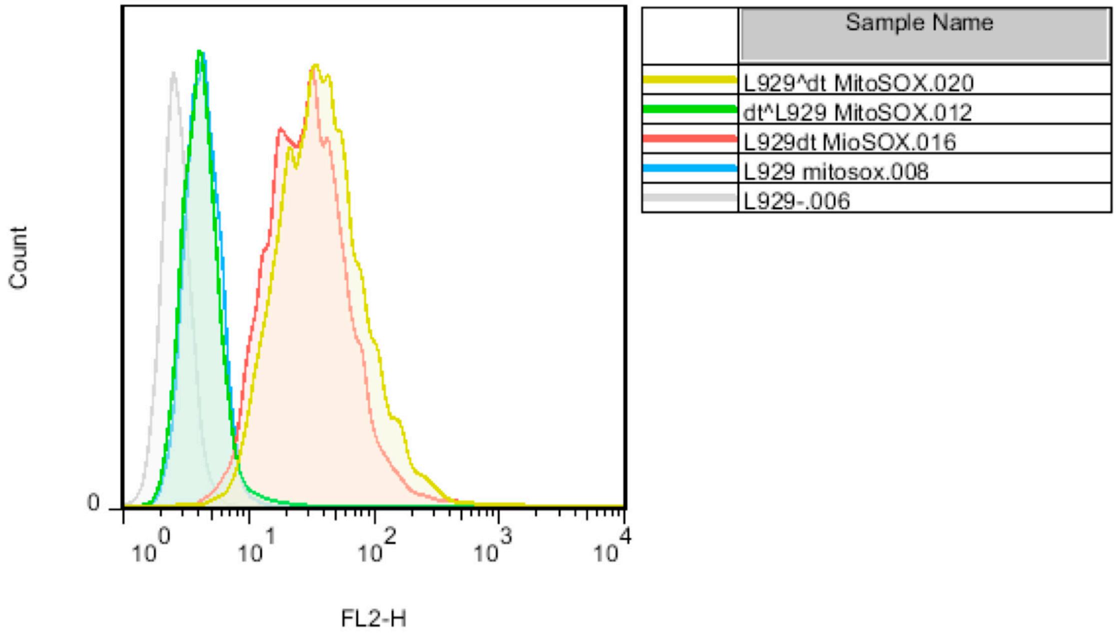

Figure S5. Determination of the amount of mitochondrial superoxide anion in basal conditions using the Mitosox method in L929 (blue histogram) or L929dt cells (red histogram) or in the mitochondrial cybrids L929dt^{L929} (green histogram) or L929^{dt} (yellow histogram).
